# Preoperative Forced-Air Warming Strategy: Is It Effective in Averting Intraoperative Hypothermia in Elderly Trauma Surgical Patients?

**DOI:** 10.7759/cureus.29305

**Published:** 2022-09-18

**Authors:** Sunil M Chataule, Amarjyoti Hazarika, Kajal jain, Rajeev Chauhan, Ankur Luthra, Shyam Meena, Sameer Aggarwal, Sameer Sethi

**Affiliations:** 1 Anaesthesia and Intensive Care, Postgraduate Institute of Medical Education and Research, Chandigarh, IND; 2 Orthopaedics, Postgraduate Institute of Medical Education and Research, Chandigarh, IND

**Keywords:** forced warm air, fracture fixation, regional anaesthesia, arthroplasty, temperature regulation, hypothermia

## Abstract

Background and objectives

Inadvertent perioperative hypothermia is expected in the elderly during central neuraxial anesthesia. We aimed to compare the incidence of intraoperative hypothermia (< 36-degree celsius) between preoperative forced-air warming for 30 minutes and non-warming groups. Also, we compared the time to develop hypothermia, perioperative shivering, duration of intraoperative active warming, metabolic acidosis, surgical site infections, coagulation derangements, and post-anesthesia care unit (PACU) stay.

Material and methodology

A total of 100 American Society of Anesthesiologists (ASA) I-III (Age > 60 years) patients scheduled to undergo femur fracture surgeries under central neuraxial anesthesia were enrolled in this single-blinded prospective randomized study. They were randomly allocated into Group A (active forced-air warming for 30 minutes in the preoperative period) and Group B (without forced-air warming). Then, patients were transferred to the operation theatre, where central neuraxial anesthesia was administered for surgery. An infrared tympanic membrane thermometer measured the core body temperature during the different study points.

Results

The intraoperative hypothermia incidences were 26.0% and 68.0% in groups A and B, respectively. The mean time for developing hypothermia was found to be 143.08 ± 26.26 min and 25.88 ± 9.25 min in groups A and B, respectively. The mean duration of intraoperative active warming was observed to be 15.6 minutes and 103.6 minutes in groups A and B, respectively. The shivering and surgical site infection (SSI) grades were lower in group A.

Conclusion

A preoperative forced-air warming strategy for 30 minutes helps in reducing the incidence of intraoperative hypothermia and shivering in elderly patients undergoing femur fracture surgeries under central neuraxial anesthesia.

## Introduction

In perioperative hypothermia, a core body temperature below 36.0^o^C occurs due to thermoregulation disruption by vasodilation induced by anesthetic drugs along with exposure to a cold environment and cleansing agents [1]. Unintentional hypothermia during the perioperative phase is common during central neuraxial anesthesia, which may be as severe as general anesthesia [2]. Perioperative hypothermia has many clinical consequences, which activate the sympathetic nervous system and results in shivering, patient discomfort, platelet dysfunction, blood coagulation dysfunction, increased vasoconstriction, increased risk of surgical site infection, increased urine output, bradycardia, and others [1].

Despite the widespread institution of preventive hypothermia strategies, perioperative hypothermia remains a significant cause of morbidity and poorer outcomes in elderly trauma victims. The incidence of intraoperative hypothermia is around 60% to 90%, and the prevalence of perioperative hypothermia is 50% to 90% [1]. The incidence of femur fractures in elderly patients has risen mainly because of trivial trauma on osteoporotic fragile bones.

In elderly patients, differences in circulation and tissue mass in the skin (especially the amount of adipose tissue that acts as insulation to protect against heat loss) and metabolically active tissues lead to more heat loss by radiation and convection methods. However, the perfusion can add up to the changes both in radiative and convective heat loss via skin. Therefore, elderly people are more prone to hypothermia during surgery [3].

Data studying perioperative prevention of hypothermia, specifically in an elderly group of patients scheduled for fractured femur surgeries under central neuraxial anesthesia, is scanty and needs further studies.

Therefore, to further advance our understanding of the care of elderly trauma victims under central neuraxial anesthesia for the prevention of perioperative hypothermia, we performed a prospective randomized clinical study to know the effect of 30 minutes of preoperative active forced-air warming on perioperative hypothermia incidence and its consequences.

## Materials and methods

This single-blinded, prospective, randomized control trial was conducted at the Post Graduate Institute of Medical Education and Research (PGIMER), Chandigarh, India, from June 2020 to June 2021. Approval was taken from the Institute Ethics Committee PGIMER (approval number NK/5905/MD/913) and it was registered with the Clinical Trial Registry of India (CTRI/2020/06/025755). Written informed consent was obtained from the patients. Patients having any metabolic disorder affecting thermoregulation, uncontrolled diabetes, thyroid disorder, recent ear infection, ASA physiological status ≥ IV, and contraindications of neuraxial anesthesia were excluded from the study.

Group allocation and randomization

**Figure 1:**
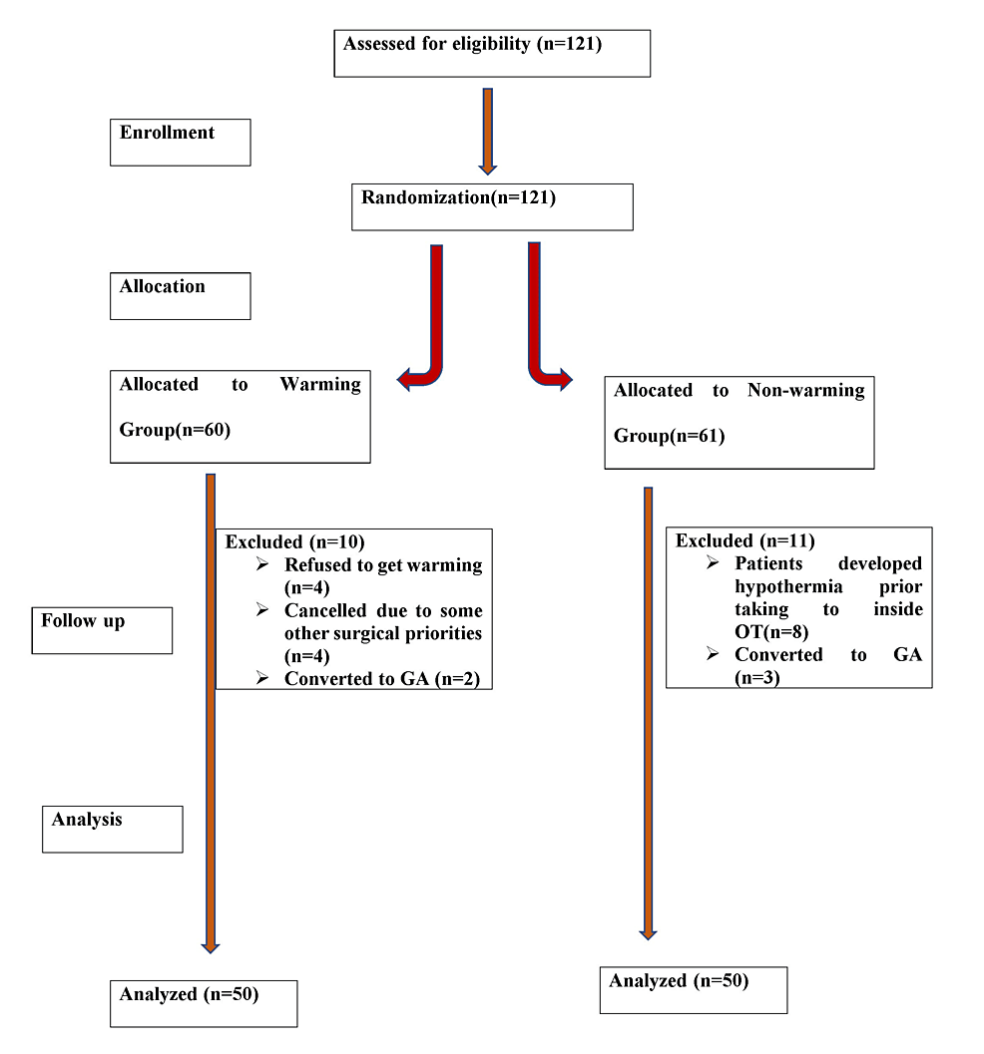
COSORT chart CONSORT: Consolidated Standards of Reporting Trials

A total of 121 patients were assessed and found eligible for the study. All eligible patients were randomized by computer-generated sealed envelope technique, into either Group A (30-minute preoperative warming group) or Group B (non-warming group). Twenty-one patients were excluded from the final analysis of our study. Finally, we enrolled/included 100 patients in this study (50 patients in each group) (Figure 1).

The observer involved in the intraoperative and postoperative period was blinded for preoperative warming status. Preoperative, intraoperative, and postoperative ambient temperatures of respective rooms were kept at a constant temperature; the preoperative and postoperative room’s ambient temperature was kept at 24.0°C, while the operative theatre’s ambient temperature was kept at 22.0°C as per our institute protocol through a centrally operating air-conditioning plant.

Methods and measurements

On arrival in the preoperative room, an intravenous cannula (preferably 18 gauge or wider) was secured, and the patient tympanic membrane temperature was noted as a baseline along with standard hemodynamic parameters. All the patients of both groups were hydrated with 500 ml of Ringer's lactate warmed at 38.0°C in the fluid warming cabinet.

As per randomization, patients in Group A were covered with an insulated blanket and warmed with forced-air warming, which was set at 40.0°C, till patient temperature reached 37.5°C ± 0.5°C. Patients were asked every five minutes about their thermal comfort and tympanic membrane temperature noted by the infrared tympanic membrane thermometer. The patients in Group B were covered with only an insulated blanket. In both groups, we ensured that before taking the patient to the operation theatre, the patient’s core body temperature was at least or above 36.0°C. The patients who developed hypothermia during the preoperative 30 minutes period were excluded from the study and kept warmed in multiple ways.

After 30 minutes of intervention and monitoring in the preoperative room, patients were shifted to the operation theatre, and shifting time was noted along with temperature and other hemodynamic parameters before shifting to the operation theatre. In the operation theatre, standard ASA monitors were attached. We covered all possible body parts during the central neuraxial anesthesia procedure. After all aseptic precautions, combined spinal-epidural was started by administrating a standard drug regimen (after confirming with an epidural test dose of lignocaine and adrenaline) preferably at L3-L4 level space as per our institute’s practice (injection bupivacaine 0.5% hyperbaric 1.5 to 2 ml + injection fentanyl 25 mcg for spinal and injection bupivacaine plain 0.5% at the rate of 5-6 ml/hourly via infusion through the epidural catheter after receding the effects of the subarachnoid block (SAB) to T12 level for maintaining of surgical anesthesia); the temperature was noted continuously along with other study parameters every 10 minutes. In the postoperative recovery room, we monitored the temperature and other hemodynamic parameters every 15 minutes till discharge from PACU after the surgery. 

After achieving an adequate level of anesthesia and appropriate surgical position, all patients were covered with blankets and were monitored for temperatures along with other hemodynamic parameters by an infrared tympanic thermometer at the study time intervals. We noted down the time when any patient developed hypothermia (<36.0^o^C) during the study period. We recorded this as the timing of developing hypothermia and active forced warm air was initiated along with other measures like using warm IV fluids in both groups.

If a patient shivered at any time during the perioperative period, it was graded by using a four-point shivering scale. Shivering was managed by active forced warming and/or by injection of 50 mg intravenous tramadol. Overall, perioperative thermal comfort in all patients was also evaluated at the time of discharge from the PACU with a 100 mm visual analog scale, i.e.,100 millimeters as insufferably hot, 0 mm as worst imaginable cold, and 50 mm as thermally neutral.

Blood samples were also collected for arterial blood gas (ABG) at baseline (before group allocation), intraoperatively, and at the end of surgery. By ABG) report, we recorded the hematocrit values and pH values, which were also compared. The prothrombin time (PT), activated partial thromboplastin time (aPTT), and platelet count were noted from the coagulation profile, at baseline, and at the end of the surgery and were compared between the two groups. The tympanic membrane temperature was measured by an infrared tympanic thermometer every 5 minutes pre-operatively, 10 minutes intraoperatively, and every 15 minutes in the postoperative period till six hours, along with other hemodynamic parameters.

The primary aim of this study was to compare the incidence of intraoperative hypothermia between the preoperative warming and non-warming group. We also aimed to compare secondary outcomes like time to develop hypothermia, perioperative shivering, duration of need of intraoperative active warming, metabolic acidosis, coagulation derangements, drop in hematocrit value, and the PACU stay duration in between groups. All the patients were followed postoperatively for adequate analgesia and stable hemodynamic parameters and were assessed for surgical site infection (SSI) (as per the Southampton wound scoring system) on day one and day three.

Statistical analysis

The sample size was calculated based on Horn et al.'s study in which 67% of the subjects developed intraoperative hypothermia and required active warming during surgery [4]. We hypothesized that in Group A, approximately 50% fewer patients will develop hypothermia during surgery (effect size 33.5%). According to power analysis, at least 50 patients were needed in each group to show a difference in intraoperative hypothermia incidences with a statistical power of 80% and CI of 95% (p<0.05).

For data analysis, IBM SPSS Statistics for Windows, Version 23.0 (Released 2015; IBM Corp., Armonk, New York) was used. The independent samples t-test was used for distributed data comparison between the two groups. Non-parametric tests in the form of the Wilcoxon test were used for abnormally distributed data. For categorical data in group comparisons, the Chi-squared test was used. Fisher’s Exact test was used if, in the contingency tables, the expected frequency was found to be less than 5 for more than 25% of the cells. Pearson’s correlation was used if the data were normally distributed, and Spearman’s correlation was used for abnormally distributed data; they are used to explore the linear correlation between two continuous variables. P < 0.05 was considered to be statistically significant.

## Results

As shown in Table 1, the demographic variables of participants, including age, gender, body mass index, and ASA status, were comparable (p>0.05). The time taken to give neuraxial block procedure, duration of anaesthesia, and duration of femur fracture surgery was also found comparable between Group A and Group B.

**Table 1:** Demographic and baseline parameters comparison between groups Values are mean ± SD or number of patients (percentages); * Significant at p<0.05; †: Wilcoxon-Mann-Whitney U Test ASA: American Society of Anesthesiologists Group B: nonwarming; Group A: preoperative forced-air warming

Parameters	Groups		p-value
	Group B (n = 50)	Group A (n = 50)	
Mean Age (Years)	70.26 ± 8.89	71.36 ± 9.43	0.5921
Age			0.6232
60 years	8 (16.0%)	9 (18.0%)	
61-70 years	21 (42.0%)	17 (34.0%)	
71-80 years	15 (30.0%)	13 (26.0%)	
81-90 years	6 (12.0%)	10 (20.0%)	
>90 years	0 (0.0%)	1 (2.0%)	
Gender			0.6852
Male	22 (44.0%)	20 (40.0%)	
Female	28 (56.0%)	30 (60.0%)	
Weight (Kg)	66.10 ± 7.65	65.96 ± 8.45	0.9391
BMI (Kg/m^2^)	23.77 ± 1.89	23.63 ± 2.15	0.8981
ASA status			0.6612
I	14 (28.0%)	18 (36.0%)	
II	27 (54.0%)	25 (50.0%)	
III	9 (18.0%)	7 (14.0%)	
Co-morbidities (present)	35 (70.0%)	33 (66.0%)	0.6682
Duration of surgery (minutes)	134.20 ± 28.93	130.60 ± 21.61	0.5811
Duration of anaesthesia (minutes)	177.00 ± 27.20	177.40 ± 23.80	0.8111
Duration of preoperative warming (minutes)*	0.00 ± 0.00	30.00 ± 0.00	<0.001 †

The association of study outcomes between groups is shown in Table 2. Intraoperative hypothermia incidence was found to be 26.0% in Group A and 68.0% in Group B.

**Table 2:** Association of the primary and secondary study outcomes between groups Values are mean ± SD or number of patients; *Significant at p<0.05; †: Wilcoxon-Mann-Whitney U Test; ‡: Chi-Squared Test; §: Fisher's Exact Test PACU: post-anaesthesia care unit; PRBCs: packed red blood cells; SSI: surgical site infection; POD: postoperative day Group B: nonwarming; Group A: preoperative forced-air warming

Parameters	Groups		p-value
	Group B (n = 50)	Group A (n = 50)	
Duration of intraoperative active warming (minutes)*	103.60 ± 76.53	15.60 ± 29.57	<0.001 †
Hypothermia (present)*	34 (68.0%)	13 (26.0%)	<0.001 ‡
Time to develop hypothermia (minutes)*	25.88 ± 9.25	143.08 ± 26.26	<0.001 †
PACU warming (Yes)*	37 (74.0%)	7 (14.0%)	<0.001 ‡
Intra-operative administered IV Fluid (mL)*	1790.00 ± 388.74	1638.00 ± 390.13	0.028 †
Number of PRBCs transfused	1.06 ± 0.84	0.94 ± 0.74	0.541 †
SSI Grade (POD 1)*			0.001 ‡
Grade 0	6 (12.0%)	20 (40.0%)	
Grade 1	44 (88.0%)	30 (60.0%)	
SSI Grade (POD 3)*			<0.001 §
Grade 0	2 (4.0%)	5 (10.0%)	
Grade 1	21 (42.0%)	43 (86.0%)	
Grade 2	27 (54.0%)	2 (4.0%)	
Shivering Grade*			<0.001 ‡
Grade 0	15 (30.0%)	44 (88.0%)	
Grade 1	35 (70.0%)	6 (12.0%)	
Thermal comfort score*	56.80 ± 5.51	52.00 ± 5.35	<0.001 †
Duration of PACU stay (minutes)*	236.40 ± 30.84	209.40 ± 36.49	<0.001 †

The mean time for developing hypothermia was found to be 143.08 ± 26.26 minutes and 25.88 ± 9.25 minutes in Group A and Group B, respectively. The mean duration of intraoperative active warming was observed to be 15.6 minutes and 103.6 minutes in Group A and Group B, respectively.

The shivering and SSI grade were lower in Group A. We didn’t find significant differences in terms of metabolic acidosis, coagulation derangements, and postoperative mean hematocrit value between the groups. The participants in Group A had a larger proportion of Shivering Grade 0, while the participants in Group B had a larger proportion of Shivering Grade 1.

As shown in Table 2, on postoperative day three, 4.0%, 42.0%, and 54.0% of the patients in Group B had SSI Grade 0, SSI Grade 1, and SSI Grade 2, respectively. On the other hand, in Group A, 10.0%, 86.0%, and 4.0% of the patients had SSI Grade 0, SSI Grade 1, and SSI Grade 2, respectively. On postoperative day three, patients in Group A had a larger proportion of SSI Grade 0 and SSI Grade 1 while participants in Group B had a larger proportion of SSI Grade 2.

**Figure 2:**
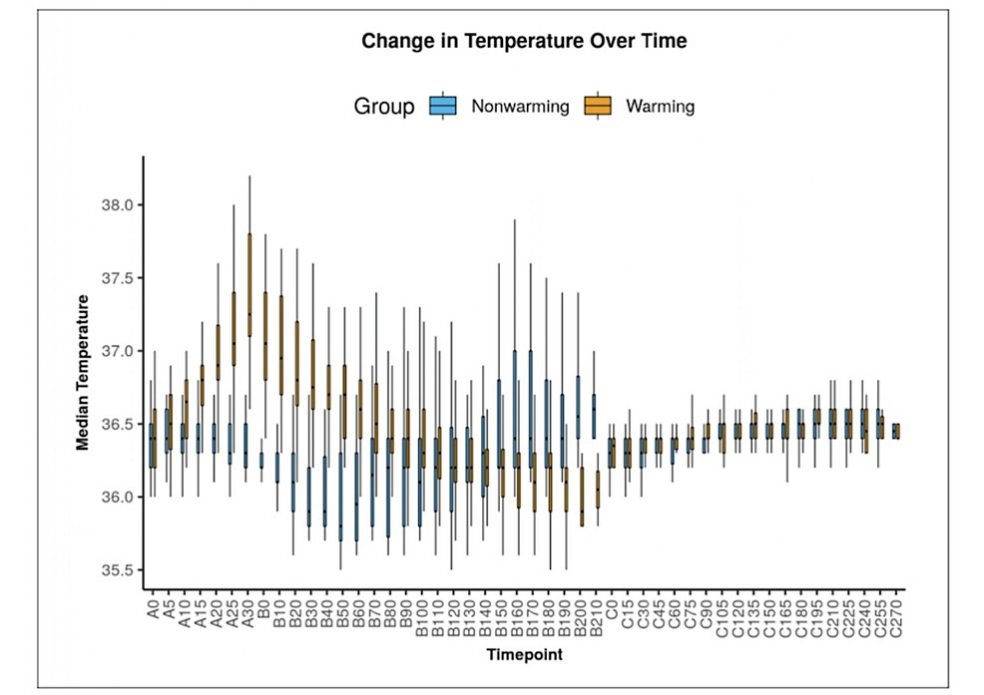
Comparison of the two groups in terms of change in Perioperative temperature over the study time A: preoperative period; B: intraoperative period; C: postoperative period Nonwarming: Group B; Warming: Group A

The distribution of temperature over different time points is depicted in Figure 2 by using the Box and Whisker plot. In each box, the median for temperature is represented by the middle horizontal line, the lower and upper bounds of the box represent the 25th and the 75th centile of temperature, and the Tukey limits for temperature at each of the time points are represented by lower and upper extent of the whiskers.

The plot depicts core body temperature was higher and falling slowly in Group A, especially in the preoperative period and first two hours of the intraoperative period as compared to Group B. We started the active warming methods immediately after noting the core body temperature below 36 degrees. It means, we required the implication of the active warming methods early and for a longer period in Group B.

**Figure 3:**
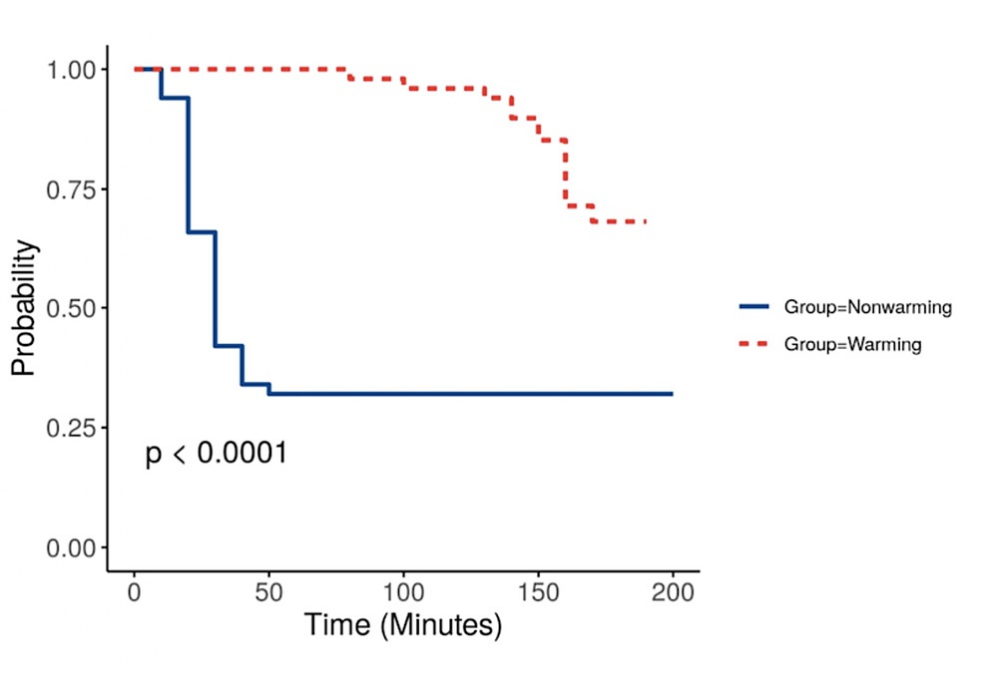
The probability of hypothermia between groups Nonwarming: Group B; Warming: Group A

The probability of hypothermia (Figure 3) is found more in Group B as compared to Group A, with a significant P value < 0.001 using the Log-rank test.

The ambient temperature in the preoperative room, surgical theatre, and recovery room was maintained at 24.0°C, 22.0°C, and 24.0°C respectively (as per institute protocol) by the central air conditioning plant strictly. None of the patients in this study had any warming-related complications like burn and hyperthermia.

## Discussion

Inadvertent intraoperative hypothermia is the most common thermal disturbance perioperatively. Its incidence varies depending on the surgical population and patients’ demographic characteristics. Old age, long duration of surgery, very severe burns, severe trauma, low preoperative temperature, and major fluid shifts intraoperatively are predisposing factors leading to a higher risk of hypothermia [5,6]. The low ambient temperature of the perioperative locations is the main culprit and that may need to be raised if hypothermia develops despite all of the prophylactic measures. Operating room lights are now often light-emitting diodes (LEDs) and they produce less heat than incandescent lights.

The present study’s result is consistent with previously published literature and found that preoperative warming reduces the incidence of hypothermia developing intraoperatively from 68% to 26%. Jo et al. studied the effect of preoperative warming by forced-air warmer for 20 minutes and non-warming on intraoperative hypothermia in elderly patients scheduled for transurethral resection of the prostate (TURP) surgery and found hypothermia incidence to be 40% vs. 59% in preoperative forced-air warming and preoperative non-warming, respectively [7]. 

Our results on shivering are also consistent with many studies. Jo et al. observed the difference in shivering at 20% vs 33% in preoperative c and preoperative non-warming, respectively [7]. In another study, Jun et al. observed a significantly lower shivering incidence (22 vs. 52%, p = 0.031) in the active preoperative warming group compared to the control group [8].

Several authors have reported that the technique of warming preoperatively by forced-air warmer decreases the hypothermia risk and prevents perioperative shivering after general anaesthesia and epidural anaesthesia and suggested that effective cutaneous warming significantly raised the body heat reserve and decreased redistribution hypothermia risk associated with general and neuraxial anaesthesia [8,9]. Analysis done previously of 19 studies with 1451 total patients recommended that preoperative forced-air warming, as a single strategy, had a significant benefit as compared to other methods of warming [10]. Many guidelines recommend at least 30 minutes of warming preoperatively to prevent perioperative hypothermia [11,12]. Horn et al. reported that in patients undergoing combined epidural and general anaesthesia, 15-minutes pre-warming between the epidural block and general anaesthesia effectively reduced perioperative hypothermia (72% vs. 6%) [12]. 

Nevertheless, the incidence of intraoperative hypothermia, even in the preoperative 30 minutes forced-air warming group (Group A) was high (26%). We believe that this could be due to age-related diminished thermoregulatory functions of the body, like vasoconstriction and shivering. In the elderly, a decrease in the release of norepinephrine and the regulation of α-adrenoreceptors reduces the vasomotor response to cold. Also, age-related loss of lean body mass decreases shivering and metabolic heat generation [13]. Advanced age is the significant predictor of hypothermia during spinal anaesthesia, except for the block level [14]. Ozaki et al. observed in their study that the vasoconstriction threshold was significantly lower (0.8°C) in the elderly patients (35.0°C +/- 0.8 °C) as compared to young patients (35.8°C +/- 0.3°C) [15]. 

The loss of heat mechanisms with central neuraxial anaesthesia are similar to those of general anaesthesia, but they also differ in important ways. Central neuraxial anaesthesia impairs autonomic temperature control like general anaesthesia [16]. During central neuraxial anaesthesia, redistribution reduces the body core temperature by approximately half as much as during general anaesthesia, so it remains the important reason for core body heat loss during the 1st hour. There is a temperature plateau phase with central neuraxial anaesthesia like general anaesthesia [17]. The part of the patient’s body below the block is unable to vasoconstrictor shiver in response to a decrease in core temperature, so heat loss is more during long cases under central neuraxial anaesthesia than under general anaesthesia [18]. Although during central neuraxial anaesthesia, heat loss from the cutaneous surface of the body to the environment is low compared to general anaesthesia due to a smaller temperature gradient, the body core temperature could fall due to the redistribution of heat. As preoperative active forced warm air reduces the temperature gradient between the core and peripheral compartments (by increasing the heat content of the peripheral thermal compartment), the quantity of heat redistribution from the core to the periphery is reduced.

We observed a statistically significant difference between the two groups in terms of the time to develop hypothermia (p = <0.001) in our study. The mean time for developing hypothermia was 25.88 minutes and 143.08 minutes in Group B and Group A, respectively. We also observed a statistically significant difference between the two groups in terms of mean duration of intraoperative active warming (p = <0.001) (103.60 minutes in Group B and 15.60 minutes in Group A). The possible explanation of the above findings is that the patients who have warmed actively (Group A) had a lesser rate of heat loss, and even maintained the euthermia for a longer period as compared to un-intervened patients (Group B). Eight patients in Group B were eliminated from the study because they developed hypothermia in the preoperative area. if we had included these patients in the study, the beneficial effect of preoperative warming would have been even more significant.

Becerra et al. observed in their study that 43% of patients had experienced shivering in the preoperative non-warming group while none of the patients develop shivering in the preoperative forced-air warming group [19]. Furthermore, there is a decrease in temperature of 0.5°C to 0.9°C due to impaired thermoregulatory response as a result of altering afferent thermal inputs, which decreases thresholds for shivering and vasoconstriction during central neuraxial anaesthesia [20].

In the present study, we found higher grade SSI in patients from the preoperative non-warming group (Group B) as compared to the preoperatively forced-air warming group (Group A). However, none of the patients from both groups required active surgical debridement or other interventions and were managed conservatively. We didn’t find many differences in terms of metabolic acidosis, coagulation derangements, and postoperative mean hematocrit value between the groups. This could be due to timely intraoperative intervention in the form of active forced-air warming and transfusion of the required blood components. Ethically also, we couldn’t allow patients to get colder. None of the patients in our study developed hypothermia beyond 35.0°C for a longer duration in both of the groups.

As per literature, hypothermia leads to an increase in the duration of hospital stay and PACU recovery time, but there are no consistent results. In a study by Kurz et al., hospital duration of stay was prolonged by 20% (2.6 days) in the hypothermic group, even after correcting the increased risk of infection [21]. Lenhardt et al. also suggested that the time to discharge from PACU is affected by perioperative hypothermia [22]. In their study, they observed that there is a significant increase in time to discharge from the PACU by 40 minutes in perioperative hypothermic patients as per the modified Aldrete and Kroulik scoring system. In our study, the mean PACU stay duration was observed statistically significant (p<0.001) 236.4 minutes and 209.40 minutes in Group B and Group A, respectively.

Limitation of the study

The absolute effect of "preoperatively forced-air warming only" couldn’t be analyzed because we gave the warmed fluid in the perioperative period to all patients, and we started the active forced-air warming just after noting the temperature beyond 36.0°C. That’s why we were not able to find out the severity of hypothermia. Ethically and clinically, we can’t allow patients to become colder for research purposes only. Another limitation is that the use of tympanic membrane temperature alone might not guarantee accurate core body temperature. We could have estimated mean body temperature and calculated the content of body heat by measuring the skin temperatures of limbs and trunks in addition to core body temperature. However, we were unable to measure the skin temperatures because applying of forced-air warming device would have interfered with the accuracy of measured skin temperatures.

## Conclusions

Preventing hypothermia is always a goal in trauma patients at all stages of care. This study demonstrates that preoperative warming with a forced-air warmer for 30 minutes prior to surgery significantly helps in the reduction of intraoperative hypothermia incidences and shivering in patients (> 60 years in age) undergoing femur fracture surgeries under neuraxial (combined spinal-epidural) anaesthesia as compared to the non-warming group. As preoperative active forced warming reduces the temperature gradient between the core and peripheral compartments by increasing the heat content of the peripheral thermal compartment, the quantity of heat redistribution from the core to the periphery is reduced.
